# Supplementary material for: Vitamin D Modified DSS-Induced Colitis in Mice via STING Signaling Pathway
Source: Biology (Basel). 2025 Jun 18;14(6):715. doi: 10.3390/biology14060715 (PMC12190092; doi:10.3390/biology14060715)
Supplement: Supplementary file 1 [file biology-14-00715-s001.zip › Supplementary Materials S1: 16s-report of gut microbiota/Groups/diff_analysis/TukeyHSD/Tukey_FAPROTAX.pdf]

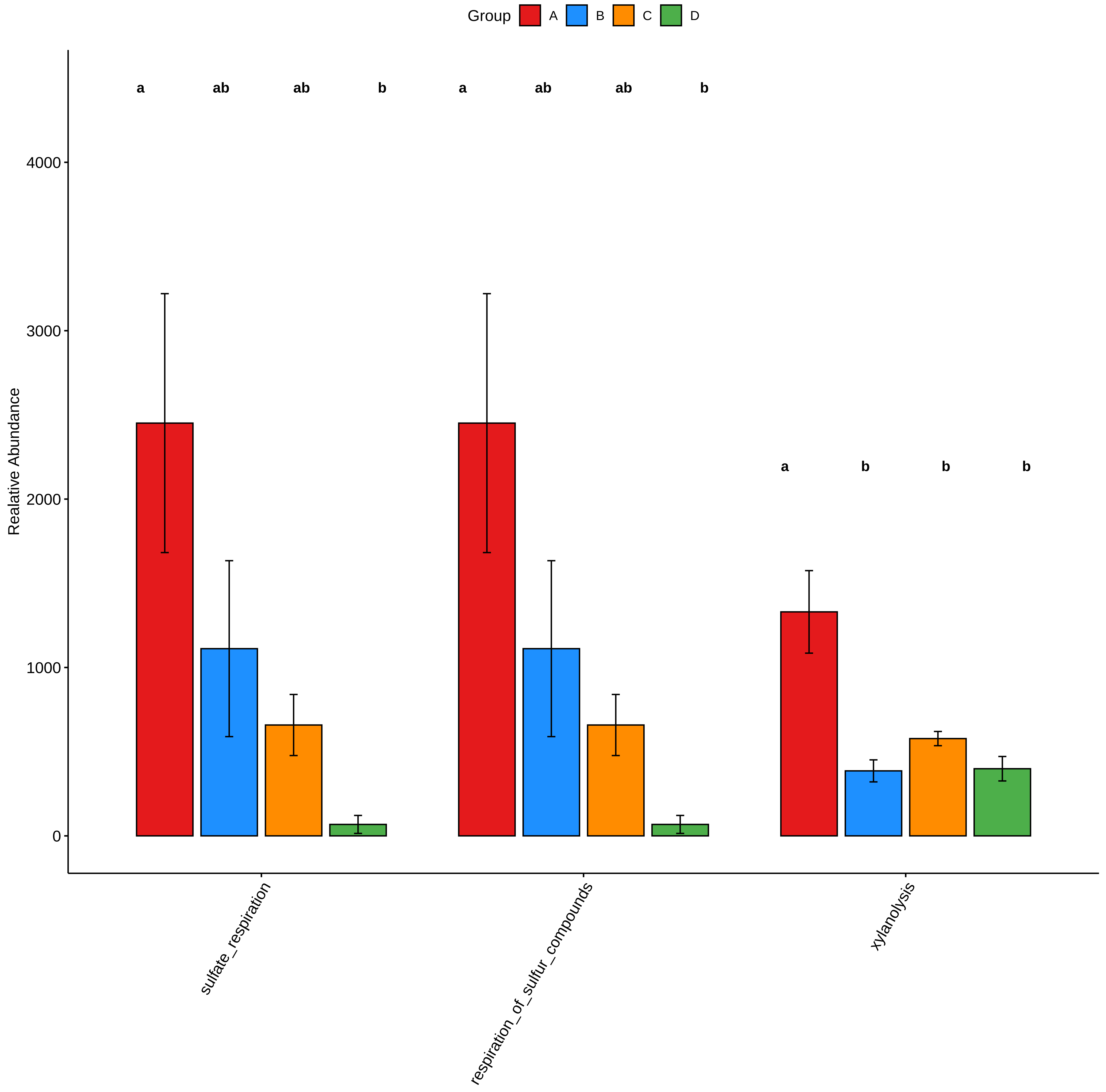
Group
A
B
C
D
a
ab
ab
b
a
ab
ab
b
a
b
b
b
Realative Abundance
4000
3000
2000
1000
0
sulfate_respiration
respiration_of_sulfur_compounds
xylanolysis
